# Pervasive Radio Mapping of Industrial Environments Using a Virtual Reality Approach

**DOI:** 10.1155/2015/701848

**Published:** 2015-06-18

**Authors:** Adrian-Valentin Nedelcu, Mihai Machedon-Pisu, Mihai Duguleana, Doru Talaba

**Affiliations:** ^1^Department of Electronics and Computers, Transilvania University of Brasov, 500036 Brasov, Romania; ^2^Department of Automotive and Transportation, Transilvania University of Brasov, 500036 Brasov, Romania

## Abstract

Wireless communications in industrial environments are seriously affected by reliability and performance issues, due to the multipath nature of obstacles within such environments. Special attention needs to be given to planning a wireless industrial network, so as to find the optimum spatial position for each of the nodes within the network, and especially for key nodes such as gateways or cluster heads. The aim of this paper is to present a pervasive radio mapping system which captures (senses) data regarding the radio spectrum, using low-cost wireless sensor nodes. This data is the input of radio mapping algorithms that generate electromagnetic propagation profiles. Such profiles are used for identifying obstacles within the environment and optimum propagation pathways. With the purpose of further optimizing the radio planning process, the authors propose a novel human-network interaction (HNI) paradigm that uses 3D virtual environments in order to display the radio maps in a natural, easy-to-perceive manner. The results of this approach illustrate its added value to the field of radio resource planning of industrial communication systems.

## 1. Introduction

Industrial automation is amongst the fields that can benefit from the ubiquity provided by IoT systems, which can be used for remotely monitoring and controlling every aspect of the industrial process. Nowadays, most industrial communication systems use hybrid wired-wireless solutions, with a preference given to wired communication protocols (e.g., Fieldbus, Modbus, Industrial Ethernet, and so on). As cost reduction has become a priority, wireless technologies tend to gain popularity in this market sector. Integrating wireless communications into a pervasive industrial environment can be a challenging and tedious endeavor, mainly due to two categories of issues/constraints.

- Performance requirements of industrial applications: reliability and real-time operation are key issues of most industrial applications; implementing a reliable wireless system is a demanding task, due to the unpredictability of the wireless transmission medium.
- Difficulties in planning a wireless network for a specific industrial environment: the key optimization task that has a crucial influence on the performance of wireless networks is choosing the best position of wireless nodes, in order to minimize the effects of fading. Due to their size and various shapes and the fact that they are mostly build of metal, industrial machinery causes multipath propagation of radio signals. They are also a source of interference, so special attention needs to be given to this issue, by implementing spectrum management techniques so as to mitigate the effects of interference.

*The main problem* this paper is addressing is the design of a radio planning system that meets the requirements and specific implementation challenges of industrial environments (i.e., large number of metal obstacles with different sizes and shapes). In order to investigate this topic, we take advantage of the pervasiveness provided by the IoT. Having a great number of nodes can help in the planning and calibration phase of the network provisioning process. These nodes can be used as spectrum sensing devices, which provide information regarding the radio spectrum that they perceive in their vicinity, by means of the RSSI (received signal strength indicator) parameter. The RSSI is an effortless, natively available resource and can be used to compute and represent a location's electromagnetic propagation map or profile. A radio propagation profile reveals the optimum electromagnetic propagation pathways but also the electromagnetic propagation obstacles.

*The secondary problem* that is investigated throughout this paper is the need of designing natural interaction interfaces between the engineers that plan the wireless industrial network and the sensing nodes that are used in this process. The human body's lack of ability to process and perceive high volume of information, and its failure to keep up with present day's data systems processing speeds suggest the necessity of high quality user interfaces for this type of applications. To compensate for these shortcomings we need to come up with new human-network interaction (HNI) systems and metaphors, especially in domains that provide the user with a high amount of data.

The main objectives of this paper are as follows:

- designing radio mapping algorithms for identifying obstacles and optimum propagation pathways within an industrial environment,
- integrating these algorithms into a radio mapping system which leverages the advantages of 3D environments and virtual reality in order to improve the interaction experience of radio planning designers,
- evaluating the performance of the radio mapping system in a real-life industrial hall.

The following sections present the activities that were performed in order to meet these objectives.  Section 2 surveys similar research activities and clearly highlights this paper's contribution to extending the body of knowledge in the field of planning industrial wireless IoT systems.  Section 3 presents the radio mapping algorithms that the authors have designed based on extensive on-site experiments and optimizations.  Section 4 presents the radio mapping system which integrates the aforementioned algorithms.  Section 5 presents the experiments that were conducted in order to assess the performance of the radio mapping approach. These experiments were performed in a different industrial hall than the one used for designing the algorithms in Section 3.  Section 6 comments the results, puts them into perspective, and underlines the correlation between the results and the initial problem statement and summarizes a number of possible future developments.

## 2. Related Work

Several studies have investigated the problem of increasing the performance of wireless communication systems operating in highly unstructured environments (i.e., industrial environments). In order to achieve this goal, one of the most challenging tasks is to identify the best propagation paths and the obstacles that influence the performance of wireless nodes. Another related task is to localize radio entities that function in such dynamic environments. For example, the relative localization of robots was achieved in [1, 2]. This idea was extended in [3], where authors propose a cooperative simultaneous localization algorithm (CSLAM) for mobile robots. The possibilities are however limitless. For example, a study proposes the localization of patients within a hospital [4].

Classic radio mapping methods which use established models of the wireless transmission medium (e.g., COST 231 [5] and Motley-Keenan model [6]) are difficult to set into practice within an industrial environment, because of the large number of obstacles (including moving obstacles) which have a variety of shapes and sizes. Also, classic methods do not take into account the interferences caused by industrial machinery and their effects on the operation of wireless networks. This is why customized methods, which combine empirical algorithms with initial calibrations, are required. Sensing nodes can be used in order to capture the “electromagnetic reality” of each specific environment.

Various radio mapping algorithms provided solutions for increasing the communication performance of wireless systems operating within industrial settings. One of these is measuring the received signal strength indicator (RSSI). Each wireless device connected to the network needs to compute the RSSI, as it needs to find the AP providing the best signal/noise ratio. In open environments, without considering interference effects, there is a direct relationship between RSSI and distance. However, in the presence of common phenomena such as reflection or refraction, this relationship needs to be adjusted.

A hybrid approach proposed in [7] attends the indoor radio mapping by developing a novel signal propagation model, called the hidden environment model (HEM). However, the algorithm is not very fast. This issue was addressed in [8] where researchers propose a system that builds the floor plan and simultaneously radio-maps the environment using a handheld laser mapping system. However, by implying the human element into the process, the method loses substance, so do other RSSI-based methods, which rely on a priori channel measurements [9, 10]. Wi-Fi propagation maps of access points (APs) are modeled deterministically or estimated using an offline human training calibration. For example, Gorce et al. [11] proposed a deterministic model based on the characteristics of the APs. Their approach uses the multiresolution frequency domain ParFlow (MR-FDPF) to calculate the radio wave propagation in indoor environments. In [10, 12], an offline training phase is performed. The work in [10] proposes a calibration scheme that is tailored to body area networks (BANs) applications. In order to improve the performance of RSSI-based radio mapping schemes, the authors of [13] represent the distribution of RSSI as a low-rank matrix and generate the dense radio map using a low-rank matrix completion method. In [14] a method based on index location is used so as to select the best radio map, among several preconstructed radio maps, for online location prediction. As one can see, RSSI-based methods may be unavailable or unreliable due to lack of previous knowledge on the environment which is implied by the offline training phase.

Other scientists propose online channel estimation techniques, based on measurements between anchor nodes [15], simulation of various propagation models [16], or using artificial intelligence algorithms such as artificial neural networks (ANNs) [17], the Hidden Markov Model (HMM) [18], or some semisupervised crowd sourced unlabeled measurements with manifold alignment [19]. However, these methods work poorly in unstructured environments or require expensive sensors [2]. Other studies explore the process of sensor relocation, in order to deal with sensor failure or respond to unexpected events [20]. However, the relocation is a complicated and expensive process, as adding mobility to network nodes makes the localization more complex and uncertain while also increasing the total costs. Accuracy is a main issue for studies such as [21]. Researches improved the localization accuracy by iteratively calculating the position probability matrixes (PPM), in order to speed up the convergence of the variance.

Another category of methods for generating radio propagation profiles is the one that uses ray tracing. Among these methods, the most used are Space Volumetric Partitioning [22], Object Distribution Technique [23], and Angular Z-Buffer [24]. The main pitfall of these methods is the computational complexity which results in high simulation times. The authors of [25] have managed to partially address this issue, obtaining simulation times which are approximately 2 times less than the other ray tracing methods. One of the main disadvantages of simulation methods based on ray tracing is that they cannot capture in real time the changes that occur in the environment. Also, from the point of view of industrial systems, ray tracing methods cannot evaluate the effect that different types of interferences have on the performance of wireless networks.

## 3. Progress beyond the State of the Art

Compared to existing radio mapping methods, the approach presented in this paper has the following salient features.

- The radio mapping algorithms designed by the authors are adapted to the requirements of industrial environments; namely, they focus on detecting the presence of machine tools that have a major impact on the performance of wireless networks.
- The algorithms are integrated into a pervasive radio planning system, based on novel human network interaction metaphors. This system incorporates key future Internet technologies, namely, an IoT spectrum sensing network, a virtual reality interaction paradigm, and a cloud-based data brokerage platform.

## 4. Designing the Radio Mapping Algorithms

**Figure 1:**
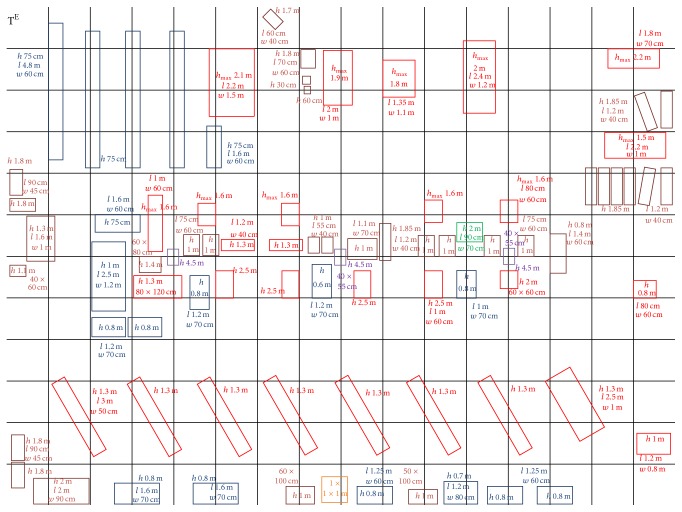
Industrial hall with various types of obstacles (test grid), *l* = obstacle length, *w* = obstacle width, *h* = obstacle height, and *h*_max⁡_ = maximum obstacle height.

Determining optimal paths for radio communication requires the detection of sources of attenuation and areas of favourable propagation. Thus one can understand the radio waves propagation medium, where phenomena such as attenuation, interference, multi-path are very common. In this respect, the quality of radio transmissions was tested in environments with severe attenuation, such as an industrial site. Based on the measurements performed in an industrial site (whose spatial configuration is presented in Figure 1), the authors have developed radio mapping algorithms for detecting obstacles and optimum propagation pathways.

### 4.1. Analyzing the Propagation Profile of an Industrial Hall

In order to show how the RSSI (received signal strength indicator) varies on the surface of the hall, the test scenario divides it into squares with a length of 1.5 m. Thus, 192 squares are obtained, made of 12 columns (the hall width is 18 m) and 16 rows (the hall length is 24 m). The RSSI value is measured in the center of each square. The emitter was positioned in one corner of the test grid. The receiver changes its position along the test grid. The height is the same for both: 0.6 m.

**Figure 2:**
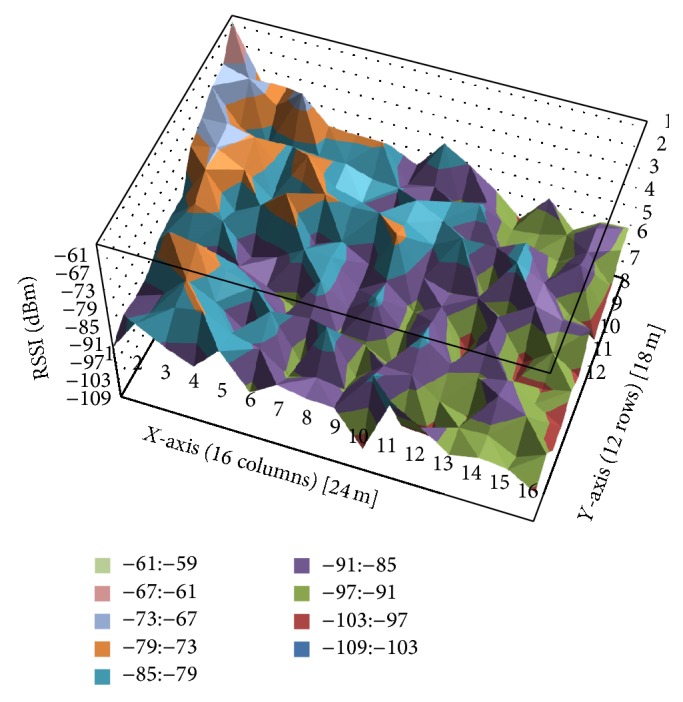
Distribution of RSSI values in the test grid.

Two sets of measurements were performed in the hall. The results for the first measurement set are shown in Table 1. The results obtained for the second measurement set are similar to the first set.  Figure 2 presents the RSSI variation throughout the grid surface.

This distribution can characterize the attenuation with distance, but it is not sufficient to describe the attenuation caused by obstructions and/or reflections due to walls and obstacles. To get a more accurate characteristic, a new approach is required. Such an approach consists in moving the emitter, in turn, in every corner of the grid, and performing four measurement sets in order to obtain this characteristic.

However, this approach is quite redundant. To reduce the computational load, the authors propose a different method. This new tactic refers to the concept of local RSSI variation and consists in averaging the RSSI values of the neighbor squares of a receiving node, whose RSSI value is compared with the average RSSI value of its neighbor nodes.

### 4.2. Algorithm for Detecting Machine Tools and Sets of Obstacles

The measurements performed in the industrial hall have revealed that there are areas around obstacles with* significant RSSI variation between neighboring zones*. These areas are usually located at the boundary between unfavorable propagation areas (close to the corners of machine tools) and favorable propagation areas (between corners there are good propagation conditions, namely, direct and short LoS to the emitter). The existence of these areas is the key to identifying attenuation sources and free (favorable) corridors between these sources. Therefore, the possibility to detect obstacles in the test grid is analyzed by determining the RSSI (local) variation around them.

Unlike the localization application of moving targets based on tomography [26], the proposed application is concerned only with stationary targets and thus it is not necessary to provide a large number of emitters and receivers, which otherwise would be quite redundant. The method for determining the RSSI variation is also different, namely,* in points*, while for detecting mobile targets a remote approach was required. The algorithm used to identify sources of attenuation is based on obtaining a* noticeable difference between the RSSI value of the area affected by potential sources of attenuation and the average RSSI value of neighbour squares*. The difference in RSSI values is measured in dBm.

The computational matrix  rssi[i][j] uses the values from Table 1. A new matrix is obtained from this matrix:  grid[i][j]. It can detect the areas affected by attenuation. For most points on the grid, these areas are determined based on Algorithm 1.

Algorithm 1 consists in the following operation: if the average RSSI value of the four neighbours of a point on the grid is greater than the RSSI value of that point then an area affected by attenuation is detected and is assigned the value 1. The remaining points of the new grid take the value of 0. For points on the grid that have only 2 or 3 neighbours, a similar procedure is used. The results obtained using this algorithm are presented in Table 2.

**Figure 3:**
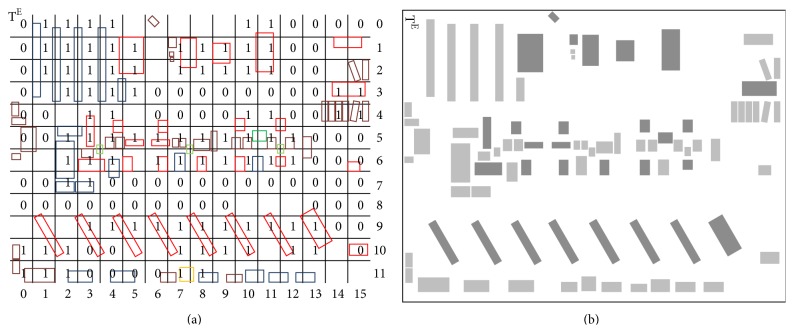
(a) Real grid (real[i][j]) with areas affected by attenuation (marked with 1) and favorable paths (marked with 0) (b) dividing obstacles in machine tools and large obstacles (dark gray) and smaller obstacles (gray).

The results from the table are compared to a real grid, as approximated in Figure 3(a), in which the central points have been marked with the corresponding values depending on the position of the receiver to the emitter (1-attenuation area). An attenuation area, marked with 1, is defined by the case in which there is one obstacle or more in that area and there is no direct LoS (up to five meters) between two nearby transmitters or at least one large obstacle (a machine tool) is in that area.

This grid provides a good indication of the main transmission corridors (marked with 0, if four zeros or more are consecutive on a horizontal or vertical line or if four ones make up a square with the same value of 0) and the areas affected by attenuation (marked with 1, if four ones or more are consecutive on a horizontal or vertical line or if four ones make up a square with the same value of 1).

For detecting areas with obstacles the following procedure is used: the real grid  real[i][j] (as seen in Figure 3) is compared with a grid obtained from  grid[i][j] which is filtered by applying Algorithm 2 rule.

### 4.3. Algorithm for Detecting Optimum Propagation Pathways

If areas with obstacles can be successfully detected, then the paths favorable to propagation, for which attenuation caused by obstacles has a small impact, should be too. The grid for favorable paths is obtained from  grid[i][j], which is filtered by applying Algorithm 3 rule.

The favorable areas are those areas for which the RSSI variation is negligible; that is, the average RSSI value of the neighbours is not greater than the RSSI value of the area with the respective neighbours. The grids obtained by comparison  success_grid_att[i][j] and success_grid_fav[i][j] are presented in Table 3. In conclusion, the radio measurements performed in the industrial hall have produced interesting results in terms of RSSI variation depending on propagation conditions. This variation has made possible the implementation of algorithms for identifying both sources of attenuation (which are usually either machine tools or sets of obstacles) and areas favourable to radio propagation.

## 5. Design and Implementation of a Pervasive Radio Mapping System

The algorithms presented in the previous section have been integrated into a pervasive radio mapping system. The system embeds a novel HNI paradigm, based on 3D virtual environments.

### 5.1. Architecture of the System

**Figure 4:**
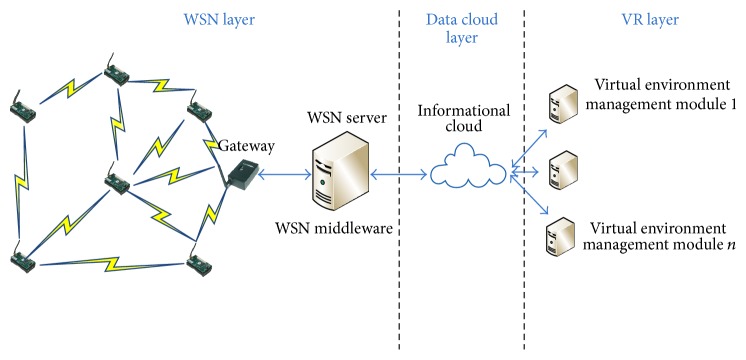
Generic architecture of the pervasive radio mapping system.

The generic architecture of our prototype radio mapping system is structured on 3 layers (as depicted in Figure 4), namely, the WSN layer, the data cloud layer, and the VR layer.

*The WSN layer* is deployed in the area of interest, with the purpose of capturing RSSI data. Based on this data the obstacle detection and radio propagation profiles are generated. Its components are

- sensor nodes, which are connected within a multihop network;
- gateway node (sink) that collects data from all nodes;
- WSN server which runs the WSN middleware; it acts as an interface between the gateway and the information cloud.

For this prototype the MICAz motes have been used. In order to implement the radio mapping algorithm, the area of interest is divided into a 16 × 12 rectangular grid, consisting of squares with a side length of 1.5 m. The MICAz motes are deployed in the center of each square in the grid. The WSN gateway is placed in the corner of the area of interest, corresponding to row 0 and column 0 of the sensor grid. The WSN gateway broadcasts test packets to all the nodes. Each node measures the RSSI of the received packet and sends a special health packet to the gateway in which the sensed RSSI is encapsulated. The WSN middleware collects the RSSI from all the sensing nodes and stores it into a sensing database. This data is made available to client applications by posting it to the data cloud layer.

The* data cloud layer* acts as a transparent sensor data sharing service that hosts the data captured by the WSN Layer and relays it to client radio mapping applications. Thus multiple virtual environments can have the same real-time perspective on the RSSI data captured by the wireless nodes. It can also enable communication between these virtual environments, hence enabling distributed users to perform a given radio planning task in a collaborative manner. For our proof of concept implementation, the ThinksSpeak data brokerage platform [27] plays the role of the data cloud layer. This platform has been chosen because its main design goal is to handle data flows generated by IoT systems in an efficient and reliable manner.

The* VR layer* is comprised of at least one* virtual environment management module (VEMM)*. The VEMM acts as a client application layer which uses RSSI data in order to build dynamic 3D radio maps of the industrial environment. When designing the VR layer, flexibility and extendibility were the main goals taken into account. Hence, it can integrate both desktop interaction paradigms, as well as immersive interaction paradigms (such as the one designed by the authors in [28]). Also, several distributed virtual environments can be interconnected through the ThinksSpeak platform, thus creating multiple replicas of the same real industrial environment. These virtual environments can use ThinksSpeak's RESTful API (or any other similar API, such as the Twitter REST API [29]) in order to synchronize their perspective of the industrial environment.

The main components of the VEMM are the following.

- VR Communication Module handles the communication between the VEMM and the data cloud layer, querying it for RSSI data by means of HTTP GET requests. It can also handle the communication with other distributed virtual environments. The implemented scenario includes one virtual environment.
- VR Perception and Interaction System provides the user with an interactive 3D environment, based on the RSSI information provided by the sensing WSN. In order to do that it implements the radio mapping algorithms presented in Section 3. The virtual environment is a replica of the industrial environment, where the WSN is deployed. The VR environment enables interaction by means of conventional interaction devices (i.e., mouse or keyboard) or through a haptic space-mouse from 3Dconnexion [30].

### 5.2. Software Implementation

#### 5.2.1. WSN Firmware

**Figure 5:**
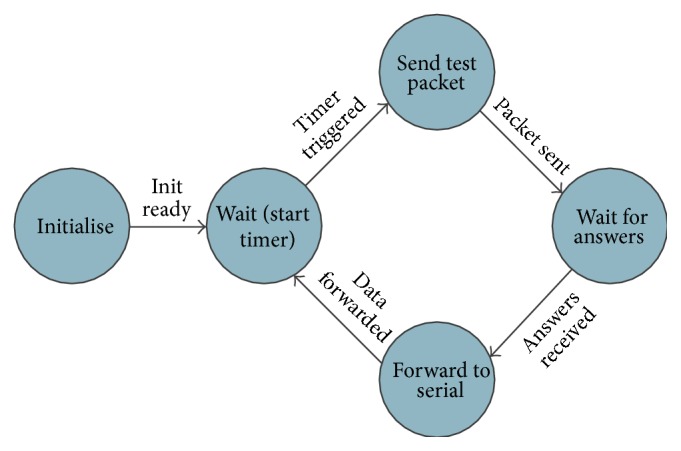
Workflow diagram of the gateway application.

The firmware that runs on the WSN sensing nodes and the WSN gateway has been implemented in nesC and it relies on the XMesh Stack [31]. The application that runs on the gateway has the following workflow (see Figure 5).

- It broadcasts test packets to all the sensing nodes.
- It waits for answers from the sensing nodes; once these answers arrived they are forwarded to a serial port, on which the WSN middleware listens.

**Figure 6:**
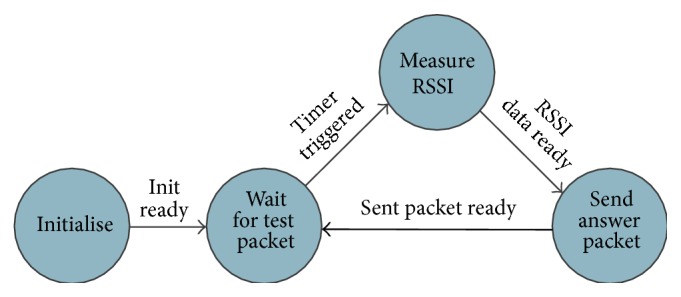
Workflow diagram of the RSSI sensing application.

The application that runs on the sensing nodes performs the following tasks (see Figure 6).

- Receive test packet from the gateway.
- Measure the RSSI of the test packet.
- Generate an answer packet that encapsulates the RSSI data.
- Send the answer data to the gateway.

#### 5.2.2. WSN Middleware

The middleware was implemented in LabVIEW. It uses a LabVIEW API for Crossbow MICAz motes [32].

The application's workflow is the following.

1. Allocate resources for a connection to a MICAz gateway, connected on a virtual serial port.
2. Create a VISA (Virtual Instruments System Architecture) connection to the gateway and start listening and retaining WSN packets.
3. Wait for packets (10.000 ms in our case).
4. Get a list of WSN mote IDs from which packets have been received.
5. Read and display the most recent packet sent by each of these nodes and extract information regarding node ID, time stamp of measurement, and measured RSSI.
6. Publish sensed RSSI data to the ThinksSpeak platform using the HTTP POST Method.
7. Stop acquiring packets and close connection to gateway.
8. Destroy assigned resources.
9. Handle MICAz specific errors.

#### 5.2.3. Virtual Environment Management Module

As stated before the VEMM has 2 main components, namely, the VR Communication Module and the VR Perception and Interaction System.

The VR Communication Module was implemented in jQuery. It manages the interaction between the WSN sensing layer and the VEMM by using the HTTP GET method in order to fetch RSSI data from the ThinkSpeak platform. The XML formatted response is parsed and the extracted RSSI data is passed on to the VR Perception and Interaction System.

The VR Perception and Interaction System has the following subcomponents.

- Camera Management Module, which handles the camera that renders the scene, based on the interaction commands provided by the user; it provides 3 types of navigation metaphors: rotating camera navigation (the camera circles around a fixed target), free camera navigation (the user can navigate to any point using the mouse), and predefined position navigation (the user is “teleported” to 6 predefined locations);
- Interaction Menu Management Module used for generating and managing the user menu, which has the following options: Navigation Options (for selecting the desired camera navigation metaphor), Visualize Maps (for visualizing the 3 types of radio maps), and Toggle (allows the user to hide/show certain parts of the virtual environment, such as the roof of the building or the industrial machinery);
- Object Selection Module;
- Object Manipulation Module;
- Scene Rendering Module;
- Radio Maps Generation Module.

In order to generate the 3D virtual environment and to implement the Perception and Interaction Module the XVR (extreme virtual reality) framework was employed [33]. The key features that made us consider XVR as the best choice for our implementation are the following:

- import 3D models from 3DSMax 4.0 or higher,
- powerful OpenGL rendering engine,
- scene-graph management,
- remote TCP and UDP connections,
- its client interface which is embedded in a HTML page and can be accessed remotely using a standard web browser.

The 3D models of the obstacles present in the analyzed industrial environment were designed in 3DStudioMax and imported as virtual objects in XVR. Thus a virtual 3D replica of the industrial environment was generated. Once this replica is constructed, the RSSI data and the algorithms presented in Section 3 are used for rendering the radio maps. These algorithms interpret raw RSSI data and give it meaning, through color coding and 3D spatial representation of propagation profiles.

The 3 types of radio maps generated by the system are presented in the following paragraphs.

**Figure 7:**
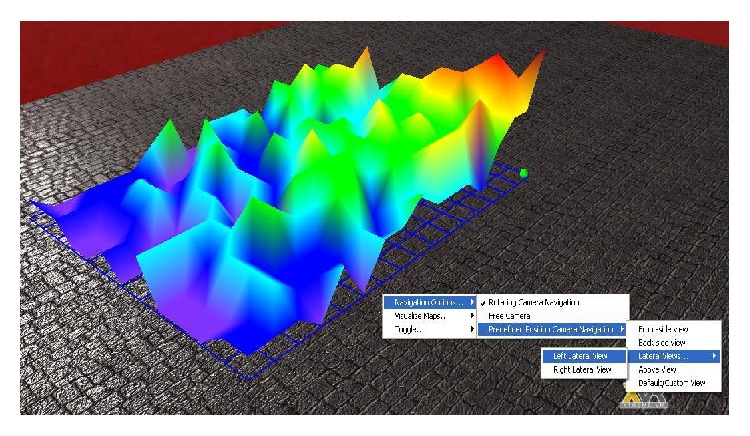
3D radio map representation of the signal's power distribution inside the indoor profile.

General radio map (Figure 7) is used for representing the signal power spatial distribution in the area of interest. The RSSI values of the signals received by each sensing node are represented in the 3D virtual environment with the appropriate color coding (red for highest RSSI values and violet for the lowest). In order to provide a better spatial overview of this distribution, the highest values of RSSI have the highest *Y*-axis coordinates. The user has the option of interpolating in-between values. In Figure 7 the green sphere from the corner corresponding to the highest power values represents the WSN gateway.

**Figure 8:**
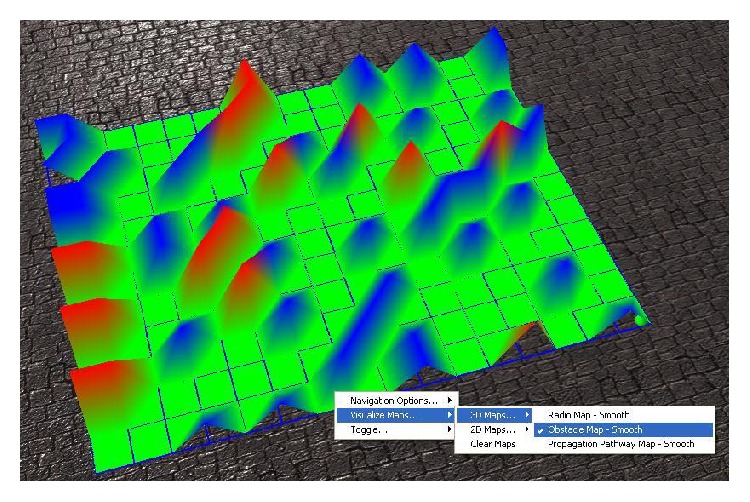
3D representation of the detected obstacles and their type, red for severe obstacles, and blue for slight obstacles.

The second representation is called “Obstacle Map,” and it represents the map of the discovered obstacles in the industrial building. The obstacles are divided into two types, according to the severity of their disturbance exerted upon the indoor radio spectrum. The more severe obstacles are color-coded red and the less severe ones are color-coded blue, while the areas that do not have obstacles are depicted as green. For 3D representations severe obstacles get a high *Y*-axis value, slight obstacles get half the *Y*-axis value that severe obstacles get, and the absence of obstacles is marked with a null *Y*-axis value. This is portrayed in Figure 8 along with Interaction Menu representation of how the user can access the Obstacle Map.

**Figure 9:**
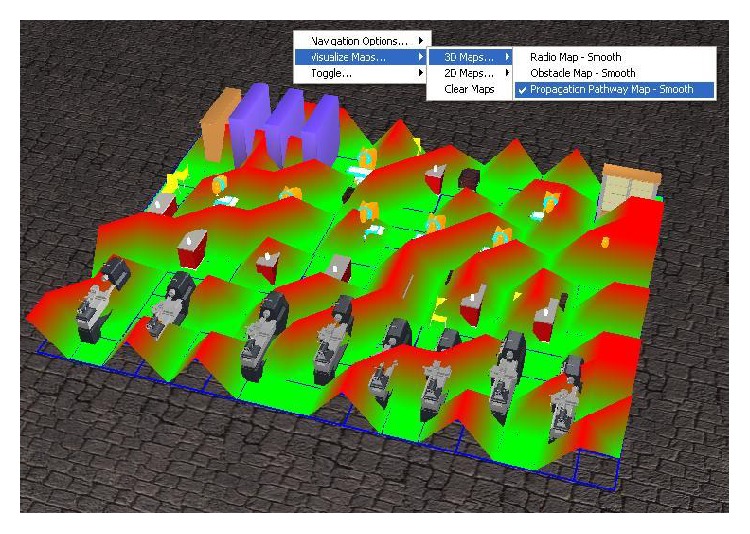
A 3D representation of the detected propagation pathways, color-coded green, and detected obstacles, color-coded red.

The third type of radio map, “Propagation Pathway Map,” is similar to “Obstacle Map,” but instead of detecting and representing obstacles, it represents the pathway upon which the signal can travel with the least perturbation possible. It identifies the pathways where the transmission should be made, color-coded green, and the locations where transmitters and receivers should be placed for optimum coverage of the industrial facility. For 3D representations obstacles get a high *Y*-axis value, and the absence of obstacles is marked with a null *Y*-axis value. The representation of this information superimposed over the actual obstacles from inside the indoor profile is represented in Figure 9. Also here one can observe the Interaction Menu's representation of how the user can access the Propagation Pathway Map.

## 6. Experiments and Results

**Figure 10:**
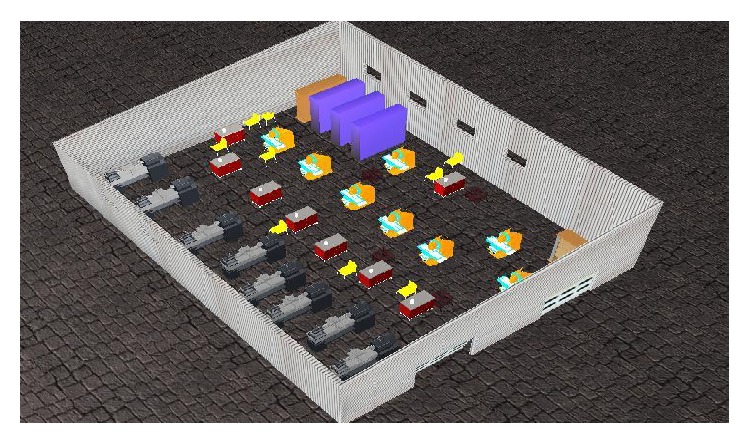
3D representation of industrial hall B.

In order to validate the pervasive radio mapping system and the algorithms that it embeds, the authors have performed a series of experiments in two industrial halls:

- industrial hall A—presented in Section 3;
- industrial hall B—a hall similar to hall A; the only major difference is in the number, position, and spatial orientation of industrial machinery (a virtual replica of hall B is presented in Figure 10).

### 6.1. Evaluating the Impact of Individual Machine Tools

In both industrial halls analyzed in the test scenarios, 3 main types of industrial machinery were identified, namely,

- 1st type: lathe (average size: 2.5 × 1.5 × 2 m),
- 2nd type: industrial drilling machines (average size: 0.6 × 0.6 × 2.5 m),
- 3rd type: workbenches (average size: 1 × 2 × 1.2 m).

To evaluate the attenuation caused by these 3 types of obstacles, the authors have measured the RSSI variation around each of them. Since RSSI varies depending on propagation conditions, the radiation pattern around a machine tool should outline the surrounding areas that are favorable to radio transmissions and also the areas affected by the presence of obstacles and unwanted reflections.

**Figure 11:**
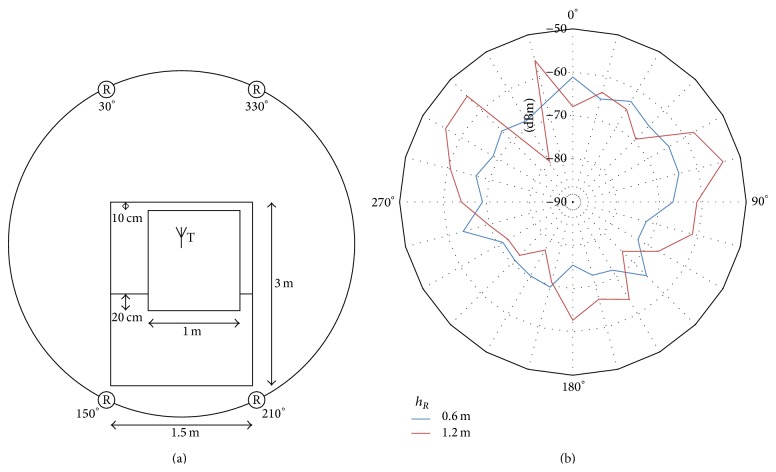
(a) Test scenario for determining the radiation pattern around the 1st type machine tool, for two receiver heights *h*_*R*_; (b) radiation pattern.

Figure 11(a) shows the test scenario for the radiation pattern based on RSSI for the 1st type of machine tool and the graphs obtained from the two measurements (Figure 11(b)). The receiver height *h*_*R*_ has two values: 0.6 m and 1.2 m. The RSSI characteristic as a function of the angle of reception can help trace these areas, either favorable to radio transmissions or unfavourable to them. At the corners of the 1st type of machine tool one can distinguish shadow areas, unfavorable to radio propagation. Such angles are 30°, 150°, 225°, and 315°. One can also see that the spaces between corners are favorable to radio propagation for the following angle intervals: 45°–120°, 165°–210°, 255°–285°, and 330°–15°.

### 6.2. Evaluating the Overall Performance of the Radio Mapping System

The two industrial halls considered in this experiment have an approximate size of 432 m^2^. The area of each hall was divided into a grid consisting of 192 equal squares. The gateway was placed in the corner of the room (i.e., row 0, column 0). A network consisting of 6 sensing nodes was deployed. At each measurement step, these nodes were moved so as to cover all the squares in the grid. The pervasive radio mapping system presented in Section 4 was utilized for capturing the RSSI data and for generating the radio maps.

**Figure 12:**
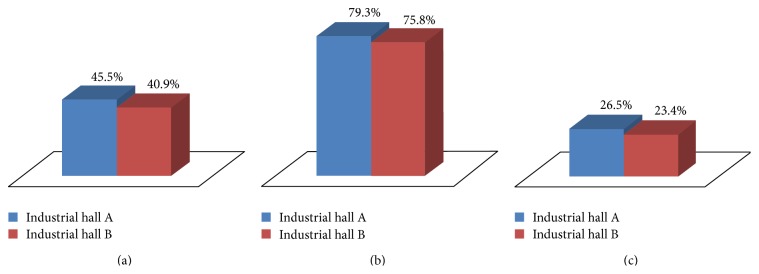
(a) Overall obstacle detection rate; (b) machine tool detection rate; (c) small obstacle detection rate.

Five sets of measurements were performed in industrial hall A. The same was done for industrial hall B. The average percentage of successfully detected obstacles is presented in Figure 12.

From Figure 12(a) one can notice that, in the case of industrial hall A, out of the 80 squares that contained obstacles, an average of 45.5% were successfully discovered, while, in the case of industrial hall B out of the 64 squares covered by obstacles, an average of 40.9% were successfully discovered. A much better result was obtained in the case of large obstacles (i.e., machine tools), for which the detection rate was larger than 75% in both scenarios (see Figure 12(b)). This performance is essential in the operation of the radio mapping system, taking into account that machine tools are the most serious cause of attenuation in an industrial environment.

For small obstacles (i.e., with a maximum size smaller than 10*λ*, which in the case of the 2480 MHz frequency used in these experiments is 1.2 m) the detection rate was around 25% (Figure 12(c)). Fortunately their impact on the performance of wireless industrial networks is less significant than that of machine tools.

Another performance metric that was used to evaluate the performance of the obstacle detection algorithm is reliability (i.e., the percentage of correctly identified obstacles, defined as the ratio between the number of correctly detected obstacles and the total number of obstacles detected).

**Figure 13:**
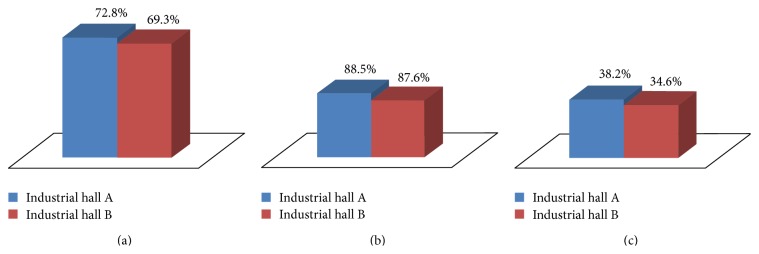
(a) Overall correct detection percentage; (b) correct detection percentage for large obstacles; (c) correct detection percentage for small objects.

In Figure 13(a), one can notice that, in the case of industrial hall A, 72.8% of the squares that were identified as containing obstacles were indeed covered by obstacles. For industrial hall B the correct detection percentage is 69.3%. For obstacles that cause a high level of attenuation the correct detection percentage was around 88% (Figure 13(b)), while for objects with a low level of attenuation it was approximately 36% (Figure 13(c)).

**Figure 14:**
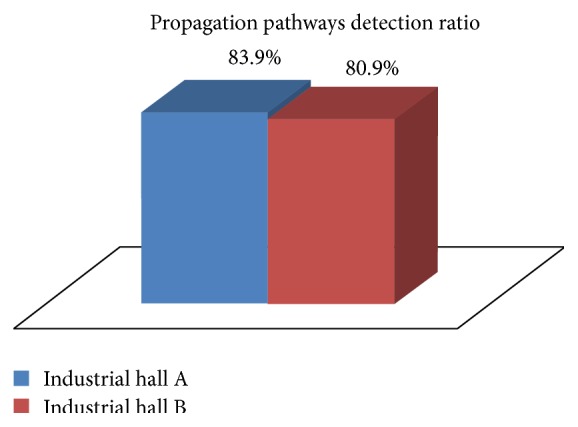
Percentage of successfully detected favorable propagation pathways.

Regarding the performance of the algorithm for detecting optimum propagation pathways, in the case of industrial hall A, out of the 112 areas which are potentially suitable for radio propagation (192 − 80 = 112 squares in the grid) an average of 83.9% were successfully detected (Figure 14). For the 2nd industrial hall considered in the experiments, out of the 128 areas that are free of obstacles, an average of 80.9% were successfully detected.

By comparing the results obtained in two different environments, one can conclude that the empirical radio mapping algorithms that were designed based on the measurements performed in industrial hall A have been successfully validated for a different scenario (i.e., industrial hall B).

## 7. Conclusions and Future Developments

This study has analysed the challenges related to the design of a pervasive radio mapping system that meets the requirements of highly unstructured industrial environments. This system uses a pervasive network of sensing nodes for capturing RSSI data, in order to compute the radio propagation profile that highlights the optimum radio propagation pathways, as well as the main obstacles within the environment.

In relation to the objectives stated at the end of Section 1, the paper's key findings and conclusions are the following.

- The radio mapping algorithms designed by the authors use the method of local RSSI variations with the purpose of identifying obstacles and favorable propagation areas. This approach has shown promising results especially in the case of large obstacles, for which the detection rate was higher than 75%. Moreover, these algorithms have shown consistent results for two different industrial environments, which proves that these methods can be integrated in a generic radio planning system.
- The authors have designed a generic architecture of a pervasive radio mapping system which integrates virtual reality technologies. The novel human-network interaction metaphors embedded in the system enhance the interaction experience of radio planning engineers. This architecture has been successfully validated through a proof-of-concept implementation, which has been tested in 2 industrial environments. This implementation shows the potential of converging two fields, namely, the Internet of Things with the Internet of 3D Environments in order to improve existing radio planning methodologies.

As a future development the authors plan to implement a fully automated RSSI sensing system. The sensing nodes will be placed on moving platforms, enabling them to sweep the entire surface of the industrial hall in order to capture RSSI data.

## Figures and Tables

**Algorithm 1:**
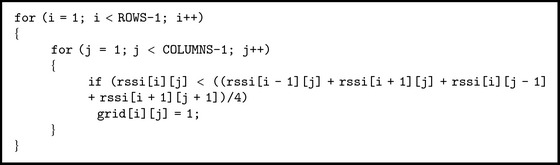


**Algorithm 2:**
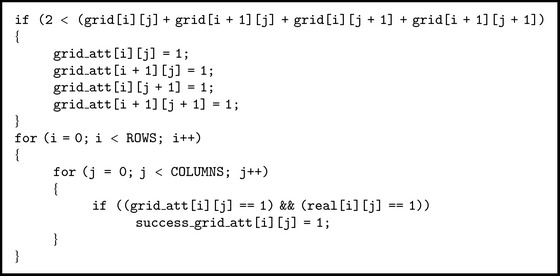


**Algorithm 3:**
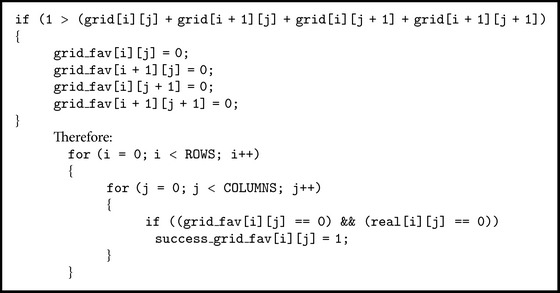


**Table 1:** First measurement set of RSSI values in the test grid (R-row, C-column).

R/C	1	2	3	4	5	6	7	8	9	10	11	12	13	14	15	16
1	−59,9	−75,8	−75,9	−82,2	−93,4	−89,2	−85,7	−89,5	−80,5	−85,3	−90,8	−97,9	−89,0	−97,0	−92,2	−91,7
2	−67,2	−69,5	−75,7	−69,8	−77,7	−78,2	−78,2	−89,6	−88	−92,1	−88,7	−95,1	−94,3	−92,1	−87,6	−92,2
3	−61,0	−78,3	−82,7	−80,1	−81	−81,1	−83,7	−84,4	−91,0	−84,1	−82,8	−94,5	−94,2	−94,1	−88,5	−97,6
4	−71,7	−71,4	−73,3	−81,6	−74,7	−82,5	−84,7	−89,7	−79,4	−83,5	−88,1	−87,5	−83,6	−92,2	−85,7	−98,8
5	−74,2	−72,5	−77,1	−83,9	−81,8	−77,9	−79,7	−79,2	−79,9	−84,7	−80,0	−97,6	−83,0	−87,2	−94,9	−101,2
6	−83,8	−73,0	−83,7	−79,7	−73,6	−87,3	−84,1	−89,0	−77,1	−88,4	−91,1	−89,8	−95,2	−87,4	−89,8	−90,1
7	−83,6	−77,3	−80,2	−89,6	−87,1	−82,2	−82,3	−90,1	−85,3	−92,5	−88,3	−100,7	−86,5	−99,1	−93,1	−98,7
8	−84,3	−85,3	−89,8	−85,7	−89,8	−94,2	−82,8	−89,4	−83,3	−92,3	−85,8	−95,9	−86,7	−97,8	−97,7	−95,6
9	−88,1	−72,7	−73,9	−80,1	−82,7	−89,9	−87,9	−91,8	−91,7	−96,5	−83,3	−89,8	−91,5	−89,7	−95	−100,1
10	−88,7	−85,8	−78,6	−83,1	−89,7	−81,0	−94,8	−80,7	−94,8	−88,2	−88,4	−94,7	−97,2	−87,	−91,1	−100,2
11	−87,6	−84,8	−80,2	−78,5	−83,4	−85,6	−92,2	−89,7	−91,2	−83,7	−97,9	−98,6	−92,6	−92,3	−96,9	−93,0
12	−91,5	−80,8	−85,8	−89,6	−81,6	−92,7	−87,4	−89,1	−89,4	−100,7	−81,8	−87,3	−93,8	−91,8	−92,8	−98,1

**Table 2:** The new grid g rid[i][j] obtained using the algorithm.

R/C	0	1	2	3	4	5	6	7	8	9	10	11	12	13	14	15
0	0	*1 *	0	1	***1***	**1**	**1**	**1**	0	0	0**1**	*1 *	0	*1 *	0	0
1	**1**	0	0**1**	0	0	0	0	*1 *	0	*1 *	0	0**1**	0	0	0	0
2	0	*1 *	*1 *	**1**	**1**	0	**1**	0	*1 *	0	0	*1 *	**1**	0**1**	0	*1 *
3	**1**	0	0	**1**	0	0**1**	**1**	*1 *	0	0	*1 *	0	**1**	*1 *	0	*1 *
4	0	0	0	*1 *	*1 *	0	0	0	0	0**1**	0	***21***	0	0	*1 *	*1 *
5	*1 *	0	*1 *	0	0	*1 *	0	*1 *	0	0**1**	*1 *	0	***21***	0	0	0
6	**1**	0	0	*1 *	**1**	0	0	0**1**	0	**1**	**1**	**0**	**1**	**0**	0	*1 *
7	0	*1 *	*1 *	**1**	**1**	**0**	**1**	0	**1**	0	**1**	*1 *	0	*1 *	**1**	0
8	*1 *	0	0	0	0	**1**	0	*1 *	0	*1 *	0	0	**1**	0	**1**	**1**
9	**1**	*1 *	0	**1**	*1 *	0	***1***	0	*1 *	0	0	*1 *	*1 *	0	0	*1 *
10	0	*1 *	0	0	0	0	*1 *	*1 *	*1 *	0	*1 *	*1 *	0	*1 *	*1 *	0
11	*1 *	0	*1 *	*1 *	0	*1 *	0	*1 *	0	*1 *	0	0	*1 *	0	0	*1 *

**Table:** (a) success_grid_att [i][j]

R/C	0	1	2	3	4	5	6	7	8	9	10	11	12	13	14	15
0	0	***1***	0	1	1	1	1	1	0	0	1	1	***1***	1	0	0
1	1	***1***	1	***1***	0	0	***1***	1	0	1	***1***	1	1	0	0	0
2	0	1	1	1	1	***1***	1	***1***	1	0	0	1	1	1	0	1
3	1	0	***1***	1	***1***	1	1	1	0	0	1	***1***	1	1	***1***	1
4	0	0	0	1	1	0	0	0	0	1	***1***	1	0	0	1	1
5	1	0	1	0	0	1	0	1	0	1	1	0	1	0	0	0
6	1	0	0	1	1	***1***	0	1	0	1	1	0	1	0	0	0
7	0	1	1	***1***	1	1	0	1	0	1	***1***	1	0	1	1	***1***
8	1	***1***	0	0	***1***	1	0	1	0	1	0	***1***	1	***1***	1	1
9	1	1	0	1	1	0	1	***1***	1	0	***1***	1	1	0	***1***	1
10	***1***	1	0	0	0	0	1	1	1	0	1	1	***1***	1	1	0
11	1	0	1	1	0	1	***1***	1	***1***	1	0	0	1	0	0	1

**Table:** (b) success_grid_fav[i][j]

R/C	0	1	2	3	4	5	6	7	8	9	10	11	12	13	14	15
0	0	1	0	1	1	1	1	1	0	0	1	1	0	***0***	0	0
1	1	0	1	0	0	0	0	1	0	***0***	0	1	1	0	0	0
2	0	1	1	1	***0***	0	1	0	***0***	0	0	1	1	***0***	0	1
3	***0***	0	0	1	0	1	1	***0***	0	0	***0***	0	1	1	0	1
4	0	0	0	1	1	0	0	0	0	***0***	0	1	0	0	1	1
5	***0***	0	***0***	0	0	***0***	0	***0***	0	1	1	0	***0***	0	0	0
6	1	0	0	1	1	0	0	1	0	1	1	0	1	0	0	0
7	0	1	***0***	0	1	***0***	0	1	0	1	0	***0***	0	1	***0***	0
8	1	0	0	0	0	1	0	1	0	***0***	0	0	1	0	***0***	1
9	1	1	0	***0***	***0***	0	1	0	1	0	0	***0***	1	0	0	1
10	0	1	0	0	0	0	1	1	1	0	1	1	0	1	1	0
11	1	0	1	1	0	***0***	0	1	0	1	0	0	1	0	0	1
